# Chloridæmia in Bright’s Disease

**Published:** 1907-03

**Authors:** 


### CHLORIDAEMIA IN BRIGHT'S DISEASE.

With the introduction of the more exact methods of physical chemistry for the lucidation of many of the intricate problems of disease, a steadily increasing conviction of the importance of the inorganic constituents of the bodily fluids has been shown, and both laboratory and clinical investigations are emphasizing the fact that many of the solutions heretofore sought in the misty realms of the autointoxication hypothesis are to be found nearer at hand in the simple disturbances of molecular pressure of the cells of the body due to variation in their inorganic constituents. In the recent illuminating work of Achard and Widal on the significance of the chlorides in the body for the healthy functional activity of the kidney we see a direct application of these newer facts of physical chemistry and their bearing on a fundamental problem of pathology.

Sodium chloride, so highly important in the human economy, is so largely because it insures the constancy of the osmotic tension, or molecular pressure, in the tissues and fluids of the body. Under normal conditions the intake and output of this salt are carefully regulated and an equilibrium is maintained. Variations in the chlorides of the body entail variations in the amount of hydration of the tissues, and if an insufficient amount of chloride is eliminated, and its ingestion is continued as usual, the tissues necessarily become overhydrated in order to keep the tension of the cells in a normal state. This results in œdema, and in the œdematous serum, as pointed out by Achard, is to be found the excess of chlorides which the body has been unable to eliminate.

In many forms of nephritis the renal epithelium is incapable of eliminating its due share of chlorides, and thus arises from this cause, in part at least, the classical œdema of that disease. This overhydration, to compensate for the retained chlorides, must needs be extensive to show in the familiar forms of localized œdema, but even in its apparent absence the invisible œdema due to hydration should never be overlooked, since in its train follow many of the dreaded symptoms of Bright's disease so universally ascribed heretofore to poisoning by uric acid or to some unknown toxines which zealous experimenters have even professed to isolate, and professed also to have obtained results from injections. Whether the term chloridæmia, now used to describe this condition, is better than the old term uræmia, does not yet appear, but that it is founded on a more definite basis in accurate observation cannot be denied, and the effects of dechloridation on the dyspnœa, vomiting, diarrhœa, headache, eclampsia, and Cheyne-Stokes respiration of a so-called uræmic attack speak in no uncertain manner concerning the significance of a diminution of chlorides in diseases of which defcetive chloride elimination on the part of the kidneys is manifest.

In the treatment if Bright's disease, then, our attention must be more accurately given to the known factors of physiology—the osmotic tension of the bodily fluids as determined by their inorganic salts, particularly by their chlorides, rather than to the misty teachings of nitrogenous autointoxication. In the dechloridation principle we arrive at a basic mensurable fact for which the science of medicine is striving. While its applications may stray in various directions into the shadowy grounds outside of the lights cast by physical chemistry, yet therapeutics is an undeniable gainer by reason of the principle.—Editorial, New York Medical Journal, February 2, 1907.

